# Epiberberine suppresses the metastasis of head and neck squamous cell carcinoma cells by regulating the MMP‐13 and JNK pathway

**DOI:** 10.1111/jcmm.17954

**Published:** 2023-09-14

**Authors:** Hsin‐Yu Ho, Mu‐Kuan Chen, Chia‐Chieh Lin, Yu‐Sheng Lo, Yi‐Ching Chuang, Ming‐Ju Hsieh

**Affiliations:** ^1^ Oral Cancer Research Center Changhua Christian Hospital Changhua Taiwan; ^2^ Department of Otorhinolaryngology, Head and Neck Surgery Changhua Christian Hospital Changhua Taiwan; ^3^ Department of Post‐Baccalaureate Medicine, College of Medicine National Chung Hsing University Taichung Taiwan; ^4^ Doctoral Program in Tissue Engineering and Regenerative Medicine, College of Medicine National Chung Hsing University Taichung Taiwan; ^5^ Graduate Institute of Biomedical Sciences China Medical University Taichung Taiwan

**Keywords:** epiberberine, head and neck squamous cell carcinoma, matrix metalloproteinase‐13, metastasis

## Abstract

Head and neck squamous cell carcinoma (HNSCC) is one of the most common histological types of head and neck cancer. Epiberberine is a potent antitumour agent for several types of cancer. This study is aimed at investigating the regulatory and molecular mechanism of epiberberine on HNSSC cell metastasis. The results showed that epiberberine inhibited the motility of Ca9‐22 and FaDu cell lines at nontoxicity doses. Moreover, the epithelial‐mesenchymal transition (EMT)‐related proteins, vimentin, snail and slug, were found suppressing after epiberberine treatments. In addition, the JNK signalling cascade and the metalloproteinase 13 (MMP‐13) expression were also found downregulated by epiberberine. In conclusion, the present study demonstrates that epiberberine suppresses cell migration and invasion by regulating the JNK pathway and MMP‐13. These results suggest that epiberberine could be a potential antimetastatic agent in HNSCC cells.

## 1 INTRODUCTION

Cancers of the head and neck, which form in the oral cavity, the pharynx, larynx, salivary glands, and the paranasal sinuses and nasal cavity, are common malignancies worldwide. The most common histological type of head and neck cancer is referred to as squamous cell carcinoma (SCC), also called head and neck squamous cell carcinoma (HNSCC), which accounts for more than 90% of cases.^1^ According to the Taiwan Cancer Registry Annual Report, the incidence of head and neck cancer was close to 45 cases per 100,000 population in 2020, with a total of 10,048 new cases and 4079 deaths diagnosed,^2^ which is higher than the global average.^3^ Surgery, chemotherapy and radiotherapy are the main approaches to the treatment of HNSCC. Although the curative rate at the early stage is high, there are still more than 50% locally advanced HNSCC patients who develop recurrence within 2 years.^4^ One of the most critical factors determining the prognosis of patients with HNSCC is the regional status of the lymph nodes. The presence of one positive lymph node can even cause a decrease in survival rates. The risk of lymph node metastasis can be predicted based on tumour differentiation, invasion size and depth, and availability of capillary lymphatics. Additionally, the likelihood of lymphatic spread increases with tumour recurrence.^5^

Cancer metastasis is involved by multiple complex processes, including extracellular matrix (ECM) remodelling, epithelial‐mesenchymal transition (EMT), extravasation, intravasation, mesenchymal‐epithelial transition (MET) and colonization.^6^ EMT or MET has been known to be a central regulator in metastasis. It regulates the reversible biochemical changes that allow certain epithelial cells to achieve a mesenchymal sensation and gives epithelial‐mesenchymal plasticity to epithelial cells.^7^ These processes can be governed by a variety of growth factors and signal pathways.^8^ Tumour cells expressing an epithelial and mesenchymal characteristic are more effective in the development of circulation, colonization of secondary sites, metastasis and resistance to chemotherapy. Thus, the development of molecularly targeted drugs is needed for antimetastatic therapy.

Epiberberine is a protoberberine alkaloid found in *Sinomenium acutum*, *Corydalis turtschaninovii* and *Coptis chinensis*. It has been discovered in multitherapeutic effect, such as anticancer, antioxidant, antiadipogenesis, antidyslipidemia and antibacterial.^9^ Binding of epiberberine to the telomeric G‐quadruplex caused the disruption of telomerase, suggesting the cancerous cell apoptosis effect of epiberberine.^10^ In gastric cancer, epiberberine inhibited tumour growth by targeting p53‐dependent mitochondria‐associated pathway in vitro and in vivo.^11^ Li et al.^12^ also found that epiberberine induced acute myeloid leukaemia cell differentiation by inhibiting lysine‐specific demethylase 1. Moreover, the antimetastasis effect of epiberberine was found in breast cancer cells through the regulating of the Wnt signalling pathway.^13^ However, the effects of epiberberine on head and neck cancer cells remain unknown. This work offers one of the first investigations into the antimetastatic mechanism of epiberberine on HNSCC.

## 2 MATERIALS AND METHODS

### 2.1 Cell culture and reagents

**FIGURE 1:**
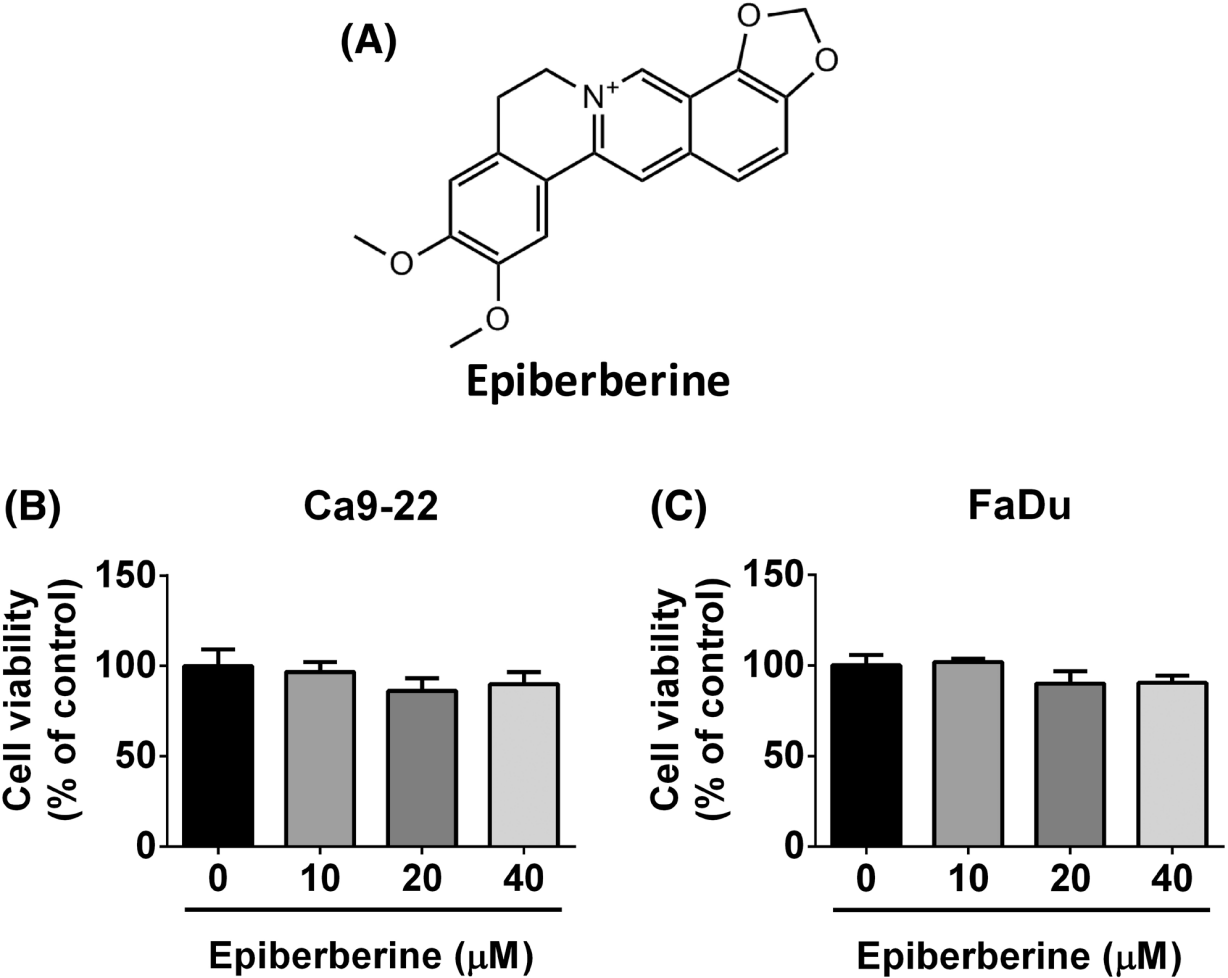
Noncytotoxicity of epiberberine at a dose less than 40 μM in HNSCC cell lines. (A) The chemical structure of epiberberine. (B, C) Cells were treated with epiberberine at different doses (0, 10, 20 and 40 μM) for 24 h. Cell viability was measured using the MTT assay. There was no difference between the vehicle group and the treatment groups in both Ca9‐22 and FaDu cells.

The HNSCC cell lines FaDu and Ca9‐22 were purchased from the Japanese collection of the Research Bioresource Cell Bank (Shinjuku, Japan). The Ca9‐22 cell line was derived from a patient with gingival carcinoma,^14^ and FaDu cell line was established from the biopsy of a patient with hypopharyngeal carcinoma.^15^ Both Ca9‐22 and FaDu cell lines are maintained in Minimum Essential Medium (Gibco) additionally with 10% fetal bovine serum (MilliporeSigma), 1% penicillin–streptomycin (Gibco) and sodium bicarbonate. All cells were cultured at 37°C humidified incubators with 5% CO_2_. Epiberberine (≥98% purity; Figure 1A) was purchased from ChemFaces. The powder of epiberberine was dissolved in dimethyl sulfoxide (DMSO) at a concentration of 40 mM as a stock reagent. The total amount of DMSO in the present experiments is less than 0.2%.

### 2.2 Cell viability assay

3‐(4,5‐dimethylthiazol‐2‐yl)‐2,5‐diphenyltetrazolium bromide (MTT; MilliporeSigma) was used for detecting cell viability. Briefly, after seeding 1 × 10^4^ cells/well of Ca9‐22 or FaDu in a 96‐well plate, different doses of epiberberine (10, 20 and 40 μM) were treated for 24 h. 0.1% DMSO was treated as vehicle group (0 μM). Subsequently, the supernatant was removed from the cells, and the MTT reagent/culture medium mixer (1:10) was incubated with cells for 3 h. Purple formazan was dissolved in DMSO and then measured using a microplate reader (BioTek) at the absorbance of 570 nm.

### 2.3 Wound‐healing assay

The wound‐healing assay is a method that measures directional cell migration in vitro.^16^ The 2‐well culture insert (ibidi GmbH) were conducted to provide the constant width of the gaps in the cell monolayer. First, placing the inserts on culture surface and seeding 3.5 × 10^3^ cells in the reservoirs. After cell attached, the inserts were removed and then the cells were treated with different doses of epiberberine (0, 10, 20 and 40 μM) for 24 h. The cell migrated ability of each group was recorded by micrography. According to the rate of migratory cells, the Ca9‐22 cell groups were recorded for 0, 6 and 24 h, while the FaDu cell groups were recorded for 0, 24 and 48 h.

### 2.4 Transwell migration and invasion assay

The transwell migration and invasion assay is used to measure the capacity of cell motility and invasiveness in vitro.^17^ Briefly, HNSCC cells were treated with different doses of epiberberine (0, 10, 20 and 40 μM) for 24 h. After placing the transwells (Greiner Bio‐One) in a 24‐well plate with fresh culture medium, cells were counted for 5 × 10^5^ cells/mL and seeded in the upper chamber of the transwells. For the transwell invasion assay, matrigel (BD Biosciences) was added to the upper chamber of the transwells before cell seeding. The transwells for the migration and invasion assay were collected at 24 h, which were then fixed and stained with 10% Giemsa stain solution (Sigma‐Aldrich). The capacity of cell migration and invasion of each group was recorded using micrography.

### 2.5 Western blot assay

To determine protein expression in epiberberine‐treated HNSCC cells, a western blotting assay was performed. Cells treated with different doses of epiberberine (0, 10, 20 and 40 μM) for 24 h were collected and lysed with RIPA buffer (MilliporeSigma) that contained protease and phosphatase inhibitors. An equally amount of samples were separated by 10% polyacrylamide gel and then transferred to polyvinylidene fluoride (PVDF) membranes (MilliporeSigma). After blocking with 5% skim milk, membranes were incubated with the indicated primary antibody at 4°C overnight. Primary antibodies against vimentin, slug, snail, matrix metalloproteinase‐13 (MMP‐13), phosphorylated extracellular signal‐related kinase (p‐ERK), ERK, phosphorylated c‐Jun N‐terminal kinase (p‐JNK), JNK, phosphorylated p38 (p‐p38) and p38 were purchased from Cell Signaling Technology; primary antibody against β‐actin was purchased from Novus Biologicals. Subsequently, secondary antibody against rabbit or mouse with peroxidase was incubated with membrane for 1 h at room temperature. The membranes were measured using a chemiluminescence photometer (ImageQuant LAS 4000 Mini; GE Healthcare Life Sciences).

### 2.6 Proteome profiler antibody array

The human protease array kit (R&D Systems Inc.; cat. ARY021B) was conducted to analyse the relative level of selected proteases. According to the manual, cell lysates were collected and incubated with membranes from the human protease array kit at 4°C overnight. After being washed with wash buffer and incubated with streptavidin–HRP solution, membranes were measured using a chemiluminescence photometer (GE Healthcare Life Sciences).

### 2.7 Bioinformatic analysis

The information from Cancer Genome Atlas (TCGA) data and the Gene Expression Omnibus (GEO) database was analysed using GraphPad Prism V6.0 (GraphPad Software, Inc.). For TCGA data, the 42 pairs of HNSCC samples (normal tissue adjacent to tumour and primary tumour) were included to determine the expression level of *MMP‐13*; for the GEO database, the 22 pairs of HNSCC samples (normal tissue adjacent to tumour and primary tumour) of GSE6631^18^ were included to determine the expression level of *MMP‐13*.

### 2.8 RNA interference assay

For investigating gene function, RNA interference (RNAi) is used to knock down the level of a particular protein.^19^ Human siRNAs for scrambled control and *MMP‐13* were purchased from Cohesion Biosciences. Ca9‐22 and FaDu cells were transfected with each siRNA using Turbofect reagent (Thermo Fisher Scientific) for 24 h. The effects of siRNA were detected by western blotting assay.

### 2.9 Statistical analysis

The statistical analysis in the present experiments was measured using GraphPad Prism V6.0 (GraphPad Software, Inc.). One‐way anova followed by Tukey's multiple comparison test is used to compare multiple groups. The Student's *t*‐test is used for comparison of two groups. A *p*‐value less than 0.05 is considered significant.

## 3 RESULTS

### 3.1 The effect of epiberberine on cell motility of HNSCC cells

**FIGURE 2:**
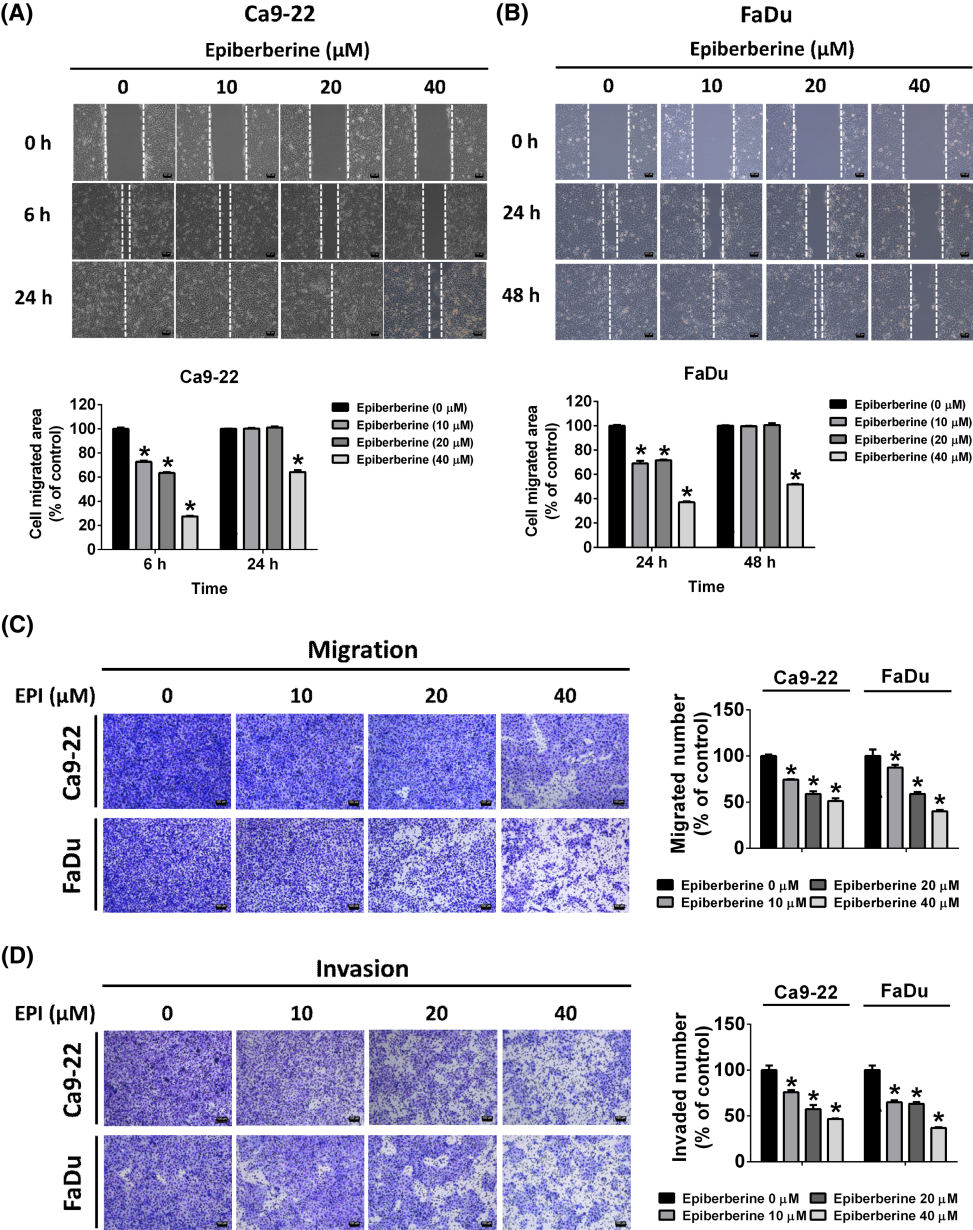
Epiberberine (EPI) suppressed migration and invasion of HNSCC cell lines. (A, B) After treatment with different doses of EPI, cell motility was recorded by microphotography at 0, 6, 24 or 48 h. (C) The number of migrated cells and (D) the number of invaded cells of Ca9‐22 and FaDu cells were counted after being treated with EPI for 24 h. Scale bar = 100 μm. *p < 0.05, compared with the vehicle group.

After treatment with different doses of epiberberine, the cell viability of HNSCC cell lines was not affected at a dose of 10 μM and slightly reduced at doses of 20 and 40 μM (Figure 1B,C). Cell toxicity did not change significantly in both Ca9‐22 and FaDu cells at epiberberine treatment doses. Based on the results, epiberberine at doses of 10, 20 and 40 μM are considered to be applicable in the following experiments. To measure the mobility of Ca9‐22 and FaDu cells, the wound‐healing assay and the transwell migration/invasion assay were analysed. As shown in Figure 2A, the area of cell migration decreased significantly in a dose‐dependent manner at 6 h and decreased at the highest dose of epiberberine at 24 h in Ca9‐22 cells, which represented that the cell mobility was decreased after epiberberine treatment. The distance of cell migration of the epiberberine‐treated FaDu cell line also showed a similar trend to the Ca9‐22 cell line at 24 and 48 h (Figure 2B). Moreover, the vertical cell mobility was measured by using a transwell migration and invasion assay. From Figure 2C,D, with the increased doses of epiberberine treatment, the stained cells were found to decrease in both Ca9‐22 and FaDu cell lines. These results demonstrated that epiberberine at doses of 10, 20 and 40 μM is effective in inhibiting cell motility in HNSCC cells.

### 3.2 The effect of epiberberine on the regulation of EMT markers of HNSCC cells

**FIGURE 3:**
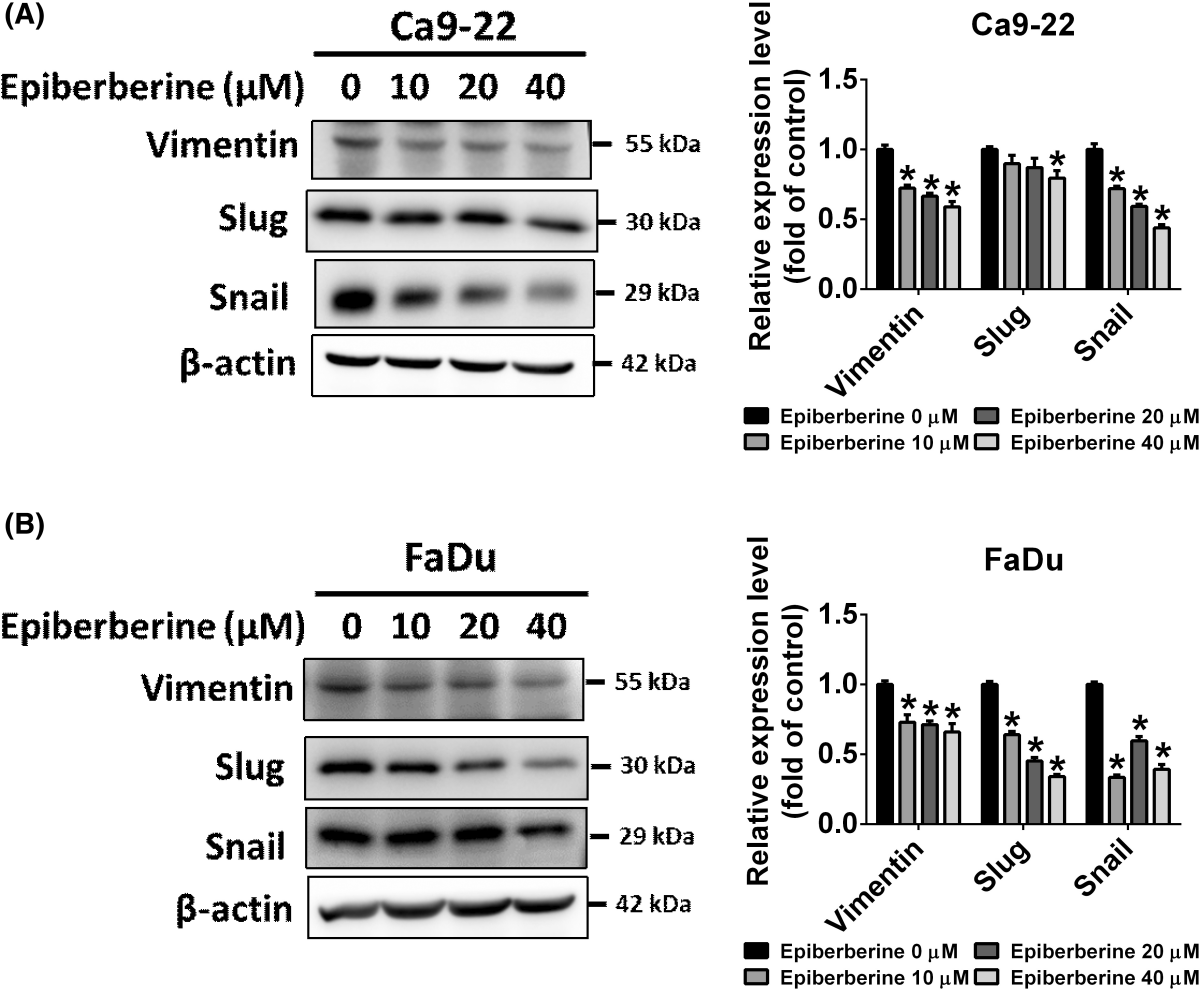
Epiberberine regulates EMT markers and transcription factors in HNSCC cell lines. (A, B) The EMT marker, vimentin and transcription factor slug and snail were measured using a western blot assay. Each relative expression level was quantified in the right panel. β‐actin as an internal control was used to adjust protein expression level. *p < 0.05, compared with the vehicle group.

EMT markers such as E‐cadherin, N‐cadherin, vimentin, fibronectin, claudin‐1 and related transcription factors (twist, snail and slug) have been found to correlate with the promotion or inhibition of cell mobility. As shown in Figure 3A,B, the intermediate filament protein, vimentin, was downregulated in epiberberine‐treated groups in both Ca9‐22 and FaDu cell lines. Furthermore, the EMT‐related transcription factors, slug and snail, were found suppressing after epiberberine treatments. The data suggested that the EMT markers may be involved in epiberberine‐inhibited cell motility.

### 3.3 MMP‐13 participates in epiberberine‐regulated cell motility

**FIGURE 4:**
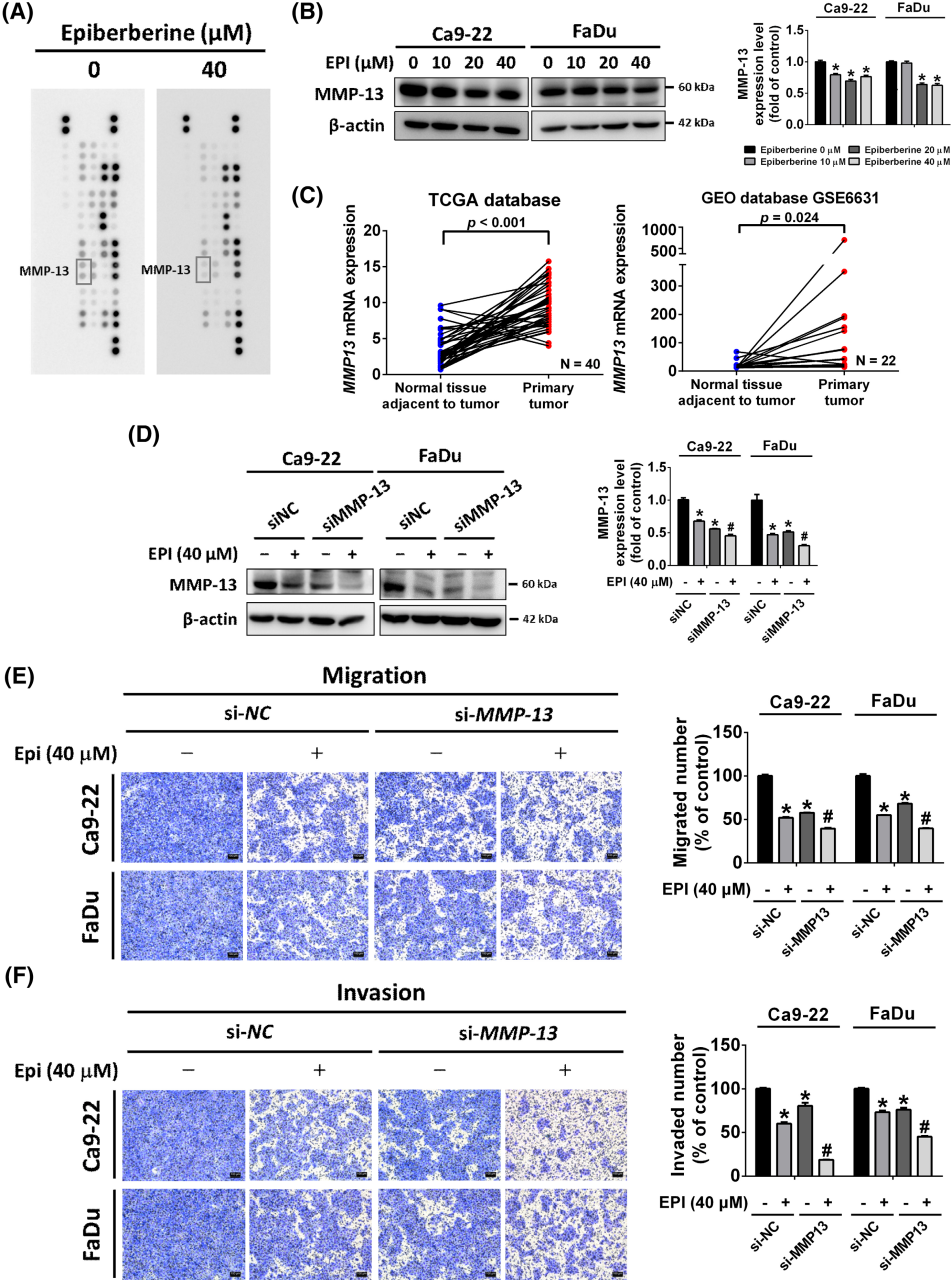
Epiberberine (EPI) suppressed cell migration and invasion by decreasing MMP‐13. (A) The Ca9‐22 cell line was treated with EPI at a dose of 40 μM for 24 h and measured using a proteome profiler antibody array kit. The expression level of MMP‐13 was labelled with a grey rectangle. (B) The protein expression level of MMP‐13 was measured using western blot after different doses of EPI treatment in Ca9‐22 and FaDu cell lines. (C) Information on the level of MMP‐13 mRNA expression was purchased from the TCGA database and the GEO database (GSE6631). The paired number of normal tissue adjacent to tumour and primary tumour was 40 in TCGA database and 22 in GSE6631, respectively. (D) Cells transfected with scramble siRNA or si*MMP‐13* followed with or without EPI treatment at 40 μM were collected to measure the expression level of the MMP‐13 protein. (E) Migration and (F) invasion assays were measured in Ca9‐22 and FaDu cells after transfected with scramble siRNA or si*MMP‐13* followed with or without EPI treatment at 40 μM. Scale bar = 100 μm. β‐actin as an internal control was used to adjust protein expression level. *p < 0.05, compared with the siNC without EPI treatment group. ^#^ *p* < 0.05, compared with the siNC with EPI treatment group.

To investigate the molecule targeting by epiberberine in the regulation of cancer cell motility, the proteome profiler antibody array was executed. After being treated with epiberberine at 40 μM, MMP‐13 was significantly reduced comparing to the vehicle group in Ca9‐22 cell line (Figure 4A). Then, the downregulation of the expression level of the MMP‐13 protein was confirmed under different doses of epiberberine treatment in both Ca9‐22 and FaDu cells, respectively (Figure 4B). Data from the TCGA and GEO database were also validated for the role of MMP‐13 in patients with HNSCC. As shown in Figure 4C, 37 of 40 pairs from the TCGA database and 19 of 22 pairs from the GSE6631 data set have found that MMP‐13 gene expression was higher in tumour tissues than in adjacent normal tissues. To clarify the role of MMP‐13 in epiberberine‐inhibited metastasis, the RNAi of MMP‐13 was then involved in epiberberine treatment at a dose of 40 μM. After transfecting with si*MMP‐13* for 48 h and following with epiberberine treatment for 24 h, the level of MMP‐13 protein was downregulated compared with the epiberberine‐alone group and the si*MMP‐13* group, respectively, in both Ca9‐22 and FaDu cell lines (Figure 4D). Moreover, the suppression of cell mobility was also found in cells, which transfected with si*MMP‐13* for 48 h and followed with epiberberine treatment for 24 h using migration and invasion assay (Figure 4E,F). The results confirmed the key role of MMP‐13 in epiberberine suppressed cell metastasis in HNSCC cells.

### 3.4 Epiberberine negatively regulated cell motility through the JNK pathway of HNSCC cells

**FIGURE 5:**
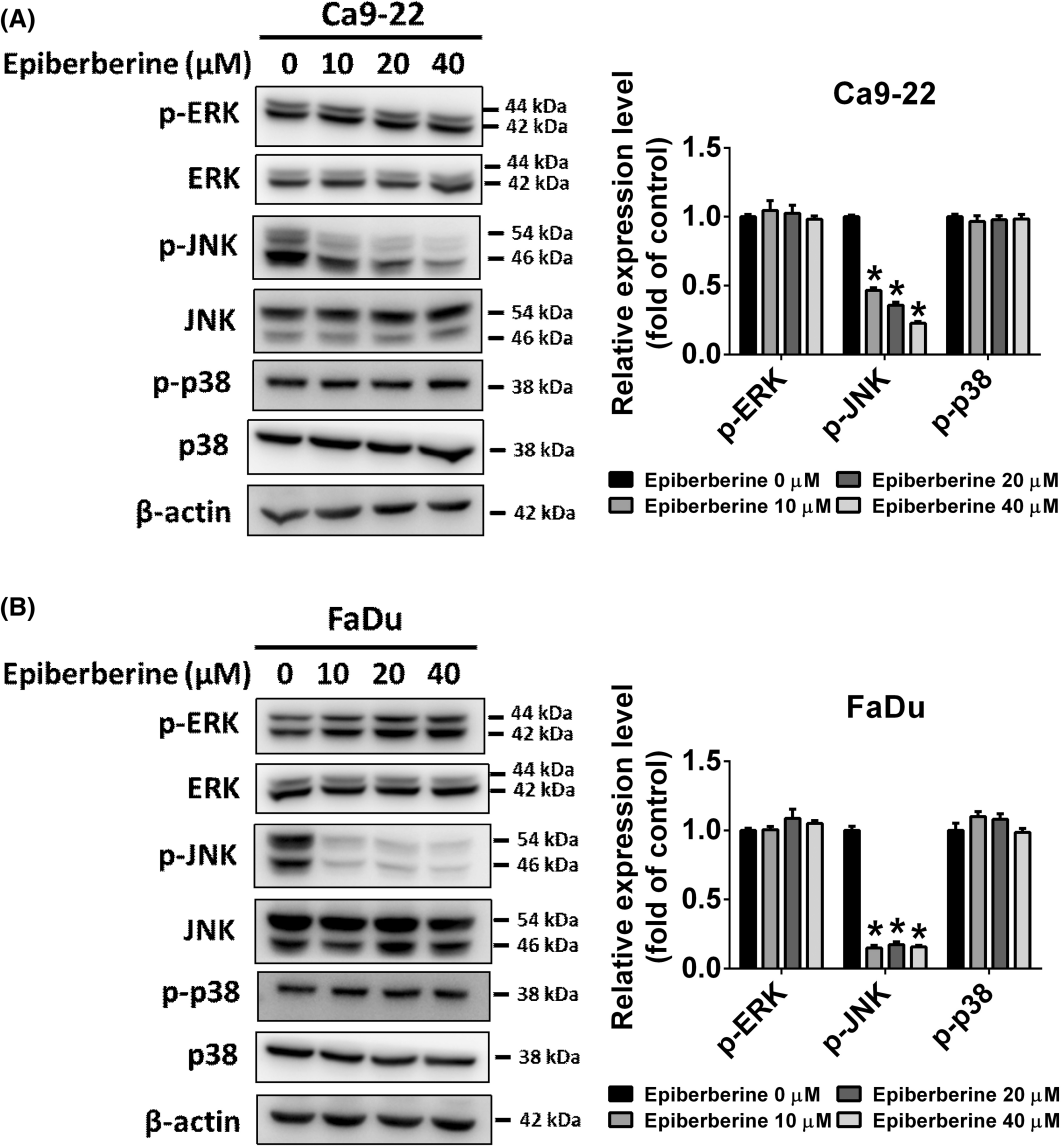
Epiberberine decreased JNK protein expression in HNSCC cell lines. (A) The protein level of ERK, JNK and p38 in Ca9‐22 and (B) FaDu cell lines was measured using a western blot assay, respectively. β‐actin as an internal control was used to adjust protein expression level. *p < 0.05, compared with the vehicle group.

**FIGURE 6:**
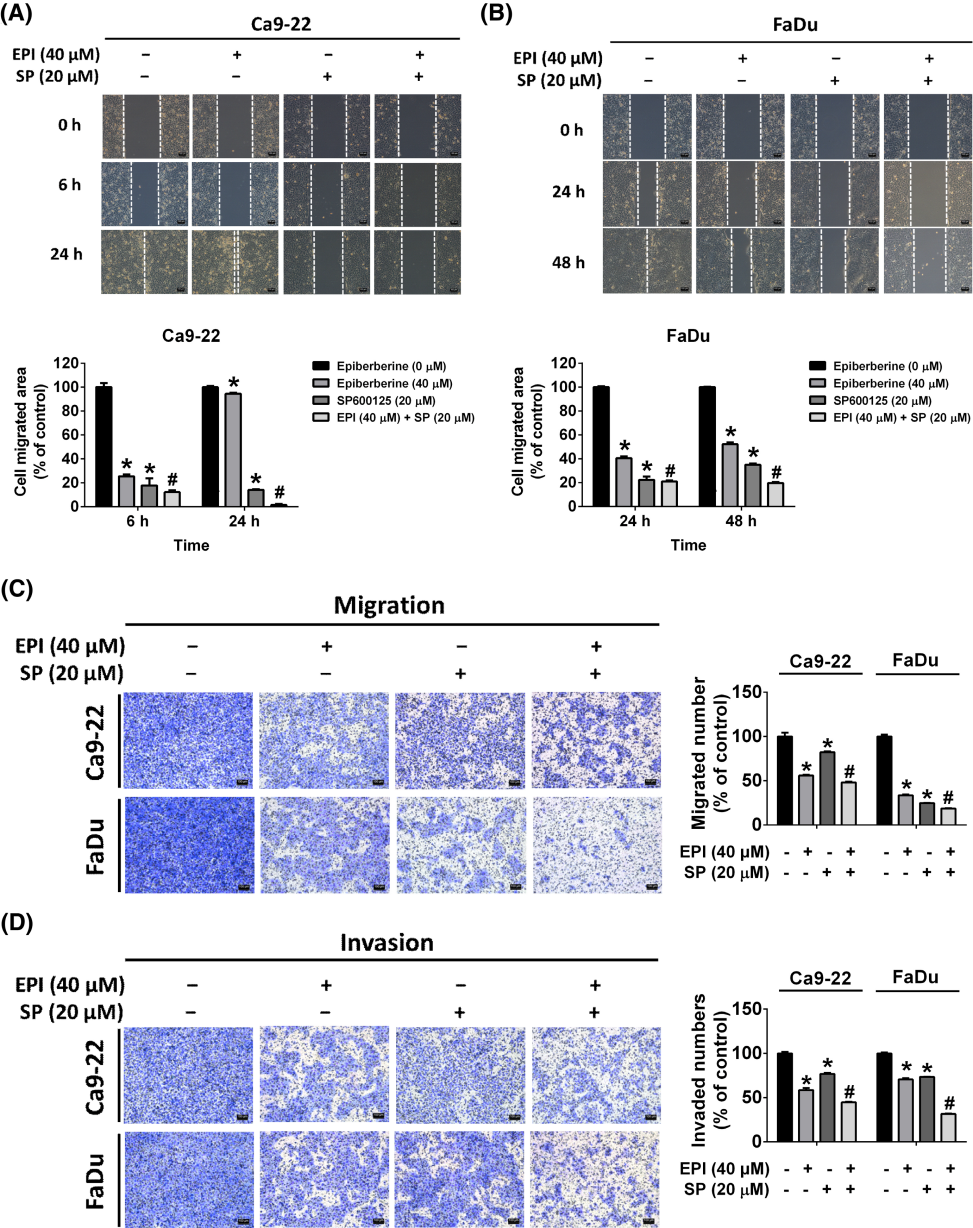
Epiberberine (EPI) suppressed cell motility through downregulation of JNK in HNSCC cells. (A, B) Cells were pretreated with SP600125 (SP) for 1 h and followed with or without EPI treatment for 24 h. Subsequently, cell motility was recorded by microphotography at 0, 6, 24 or 48 h. (C) The number of migrated and (D) invaded cells was counted after the treatment with SP, EPI or both for 24 h. Scale bar = 100 μm. *p < 0.05, compared with the vehicle group. ^#^ *p* < 0.05, compared with the EPI (40 μM) treatment group.

Mitogen‐activated protein kinases (MAPKs), including the inactive form and catalytically phosphorylated forms, are signal‐transducted kinases that are involved in cellular response. The protein levels of ERK, JNK and p38 were detected using a western blot assay. As shown in Figure 5A,B, the phosphorylated form of ERK and p38 did not change after treatment with different doses of epiberberine. However, the phosphorylated form of JNK was significantly reduced after epiberberine treatment in Ca9‐22 and FaDu cells. To further investigate whether the JNK signalling pathway participated in epiberberine‐suppressed cell migration and invasion, the specific inhibitor of JNK (SP600125) was used. Cells were treated with SP600125 at a dose of 20 μM for 1 h and then co‐treated with or without epiberberine at a dose of 40 μM. The cell migrated area of the vehicle, epiberberine‐alone, SP600125‐alone and SP600125 combined with epiberberine groups were detected using the wound‐healing assay (Figure 6A,B). The cell migrated area was found to decrease in the epiberberine‐alone and SP600125‐alone groups compared with the vehicle group, while the SP600125 combined with the epiberberine group had the lowest migrated area among four groups. Furthermore, the number of cell migration and invasion of vehicle, epiberberine‐alone, SP600125‐alone and SP600125 combined with epiberberine groups measured by the transwell assay showed a similar trend with Figure 6A,B in both Ca9‐22 and FaDu cell lines (Figure 6C,D).

## 4 DISCUSSION

The present study is designed to investigate the regulatory and molecular mechanisms of epiberberine on cell motility of HNSCC cells. The results indicate that epiberberine inhibited cell motility at nontoxicity doses in Ca9‐22 and FaDu cell lines. The EMT markers and related transcription factors were regulated by epiberberine. The combination of JNK inhibitor or *MMP‐13* siRNA with epiberberine also confirmed the role of the MMP‐13 and JNK pathway in cell motility. Epiberberine has been indicated as a low‐toxicity small molecule with multiple targets on several activities. Choi et al.^20^ have mentioned that epiberberine had no cytotoxicity at a dose of 50 μM in 3T3‐L1 preadipocytes. The IC_50_ value of epiberberine in gastric cancer cell line MKN‐45 and the hepatocellular carcinoma cell line HepG2 is 39.7 μM and 120.58 μg/mL, respectively.^11^, ^21^ Our present data revealed that epiberberine under a dose of 40 μM showed no cytotoxicity but inhibition of cell motility in HNSCC cell lines. This evidence shows that the effective condition of epiberberine is relatively safe between different cell types.

EMT is a preserved development programme involved in carcinogenesis that enhances the movement, invasion and resistance of apoptotic stimuli and confers metastatic properties in cancer cells.^22^ Epithelial cells with limited migratory potential can be identified by a variety of cell surface markers, especially E‐cadherin, but also cytokeratins, and ZO‐1.^23^ Mesenchymal cells, on the contrary, with the characteristic of front rear polarity and migratory potential, can be recognized by cell surface markers such as N‐cadherin, fibronectin and vimentin.^24^ Moreover, a number of articles in the literature indicated that snail and slug transcription factors are capable of promoting the EMT process by downregulating target genes.^25^, ^26^ Xu et al.^27^ also indicated that snail and MAPK1/slug/vimentin feedback loop are keys to suppress EMT in bladder cancer. As a natural compound, berberine has been reported to suppress EMT through upregulated epithelial markers and downregulated mesenchymal markers in gastric cancer and breast cancer.^13^, ^28^ 8‐Oxo‐epiberberine, the epiberberine analogues, showed suppression on TGF‐β1‐induced EMT.^29^ Similar to these findings, the present study shows that epiberberine inhibited vimentin, snail and slug in HNSCC cells. Our data add another evidence on the EMT suppression of berberine analogue.

MMPs belong to a family of zinc‐dependent proteinases that are capable of promoting metastasis by remodelling surrounding ECM proteins during tumour progression.^30^ MMP‐13, also called collagenase‐3, is active against a number of ECM components. It has been shown that MMP‐13 is overexpressed during the cancer metastasis process in various tumour cells, including breast cancer, colorectal cancer, papillary thyroid carcinoma, laryngeal cancer and head and neck cancer.^30^, ^31^, ^32^, ^33^, ^34^ Moreover, a number of studies indicate that downregulation of MMP‐13 is correlated with tumour metastatic suppression.^34^, ^35^ Here, the protease array discovered that epiberberine lowers MMP‐13 expression. Furthermore, the TCGA and GEO databases demonstrated that MMP‐13 expression is higher in tumour tissues than in adjacent normal tissues. We also discovered that epiberberine inhibited HNSCC metastasis through the inhibition of MMP‐13, suggesting that MMP‐13 may be a potential target to against cancer metastasis.

The MAPK cascade is known to correlate highly with tumour metastasis.^36^, ^37^ The results of the present study show that epiberberine inhibits HNSCC metastasis by suppressing JNK pathway. The finding confirms those of earlier studies that natural compounds inhibited cell motility by JNK regulation in head and neck cancer, for instance, phenethyl isothiocyanate,^38^ melatonin^39^ and kaempferol.^40^ Furthermore, MAPKs pathway, including ERK, JNK and p38, has been shown to be involved in MMP‐activated tumour metastasis in HNSCC.^41^ Zeng et al.^42^ also noted that the JNK pathway is capable of inhibiting MMP‐13‐regulated metastasis. However, given the study design, the relationship between epiberberine‐modulated JNK and MMP‐13 in HNSCC cells remains unknown. Further research is needed to explore the underlying mechanism of epiberberine‐regulated JNK/MMP‐13 and add an animal study to confirm the effect of epiberberine in vivo. Taken together, our observations show that epiberberine could be a potential antimetastatic agent on HNSCC cells by modulating the JNK pathway and MMP‐13 expression.

## 5 CONCLUSION

The present study demonstrates that epiberberine reduced Ca9‐22 and FaDu cell metastasis by inhibiting the JNK signalling cascade, EMT‐related proteins and MMP‐13 expression. Based on this study, we have first documented that epiberberine inhibits metastasis by targeting MMP‐13 in head and neck cancer cells. These findings provide new insights into the therapeutic activity of epiberberine in metastatic head and neck cancer.

## AUTHOR CONTRIBUTIONS

**Hsin‐Yu Ho:** Methodology (equal); software (equal); writing – original draft (lead). **Mu‐Kuan Chen:** Conceptualization (equal). **Chia‐Chieh Lin:** Methodology (equal); software (equal). **Yu‐Sheng Lo:** Methodology (equal); software (equal). **Yi‐Ching Chuang:** Methodology (equal); software (equal). **Ming‐Ju Hsieh:** Conceptualization (equal); writing – review and editing (lead).

## CONFLICT OF INTEREST STATEMENT

The authors declare that there are no conflicts of interest.

## Data Availability

The data used to support the findings of this study are available from the corresponding author upon request.
